# Magnetic resonance imaging findings in placenta accreta spectrum disorders: pictorial essay

**DOI:** 10.1590/0100-3984.2021.0115

**Published:** 2022

**Authors:** Natália Henz Concatto, Stephanie Sander Westphalen, Rubia Vanceta, Alice Schuch, Gustavo Felipe Luersen, Caroline Lorenzoni Almeida Ghezzi

**Affiliations:** 1 Hospital Moinhos de Vento, Porto Alegre, RS, Brasil.; 2 Hospital Cruz Vermelha, Lisboa, Portugal.

**Keywords:** Placenta accreta, Placenta diseases, Magnetic resonance imaging, Placenta acreta, Doenças placentárias, Ressonância magnética

## Abstract

Placenta accreta spectrum disorders are characterized by abnormal adhesion of the placenta that can be subdivided into three categories according to degree of invasion: placenta accreta (passing through the decidua basalis and adhering to the myometrium); placenta increta (penetrating the myometrium); and placenta percreta (invading the uterine serosa or adjacent tissues or organs). The incidence of placenta accreta has increased significantly in recent decades, mainly because of an increase in the rates of cesarean section, which is its main risk factor. Accurate prenatal identification makes it possible to institute the ideal treatment with a multidisciplinary team, significantly minimizing maternal morbidity and mortality. The examinations of choice are ultrasound and magnetic resonance imaging (MRI). When the ultrasound evaluation is inconclusive, as well as when the patient has risk factors for the condition or the placenta is in a posterior location, MRI is indicated. In cases of placental invasion of the adjacent pelvic organs, MRI is also preferable because it provides a broader field of view, which improves surgical planning. Numerous features of placenta accreta spectrum disorders are discernible on MRI, including dark intraplacental bands, uterine bulging, and heterogeneous placenta. Knowledge of these findings and the combination of two or more of them increase confidence in the diagnosis.

## Abstract (pt)

Acretismo placentário é uma condição caracterizada pela implantação anormal da placenta, que pode ser subdividida em três espectros de acordo com o seu grau de invasão: placenta acreta (ultrapassa a decídua basal e adere ao miométrio), placenta increta (penetra o miométrio) e placenta percreta (invasão da serosa uterina ou de tecidos/órgãos adjacentes). A incidência de acretismo placentário aumentou significativamente nas últimas décadas, principalmente em função da elevação das taxas de cesarianas, sendo este o seu principal fator de risco. A sua identificação pré-natal precisa permite um tratamento ideal com equipe multidisciplinar, minimizando significativamente a morbimortalidade materna. Os exames de escolha são a ultrassonografia e a ressonância magnética (RM), sendo a RM um método complementar indicado quando a avaliação ultrassonográfica é duvidosa, para pacientes com fatores de risco para acretismo placentário ou quando a placenta tem localização posterior. A RM é preferível também para avaliar invasão de órgãos adjacentes, oferecendo um campo de visão mais amplo, o que melhora o planejamento cirúrgico. Diversas características na RM são descritas no acretismo placentário, incluindo bandas hipointensas em T2 intraplacentárias, protuberância uterina anormal e heterogeneidade placentária. O conhecimento desses achados e a combinação de mais de um critério aumentam a confiabilidade do diagnóstico.

## INTRODUCTION

Placenta accreta spectrum disorders are characterized by abnormal placental implantation when chorionic villi invade the myometrium through a defect in the decidua basalis^(1)^. It can be divided into three categories according to the degree of invasion^(2-4)^: placenta accreta (adherence to the myometrium); placenta increta (penetration of the myometrium); and placenta percreta (invasion of the uterus serosa or adjacent tissues or organs).

The incidence of placenta accreta spectrum disorders has increased significantly in recent decades, mainly because of an increase in the number of cesarean sections performed^(4,5)^. The main risk factors for disorder of this type are a history of surgery, especially cesarean section, and placenta previa^(1)^. Other risk factors include having undergone hysteroscopy or assisted reproduction techniques, as well as advanced age, multiparity, and postpartum endometritis^(4,6,7)^.

Placenta accreta spectrum disorders are associated with a significant increase in maternal morbidity and mortality^(8,9)^. Accurate prenatal identification allows the ideal treatment to be instituted, the examinations of choice being ultrasound and magnetic resonance imaging (MRI). The main indications for MRI are inconclusive ultrasound findings, the presence of risk factors for placenta accreta/increta/percreta, and a posterior placental location. The ideal period for an MRI evaluation is between 28 and 32 weeks of gestation^(1,10-12)^.

## PROTOCOL FOR PLACENTAL MRI

Placental MRI examinations should be performed in a 1.5-T or 3.0-T scanner. The patient should be in the supine position with a moderately full bladder, which optimizes visualization, especially in cases of suspected placenta percreta^(13)^. The basic MRI sequences that provide images rapidly are gradient-echo and spin-echo sequences, such as single-shot fast spin-echo sequences, true fast imaging with steady-state precession (TrueFISP) sequences, and fast imaging employing steady-state acquisition sequences, all of which reduce maternal and fetal motion artifacts. Breath-holding should be used when possible^(9,11)^. Diffusion-weighted imaging (DWI) is a relatively new technique to assess placenta accreta spectrum disorders, being used as an ancillary tool to assess placental invasion, and can be useful to define the interface between the placenta and the myometrium^(14)^. The total scan time for the examination is 25-35 min, and a radiologist should be present during the examination to guide the technician in case an additional plane perpendicular to the myometrium-placenta interface or the myometrium-bladder interface is needed in order to determine the exact site of the placenta accreta spectrum disorder^(9,13)^. The use of gadolinium contrast medium should be avoided, because it has been associated with an increased risk of rheumatologic diseases, inflammatory diseases, and infiltrative skin conditions in children with a history of intrauterine exposure to contrast, as well as with an increased incidence of stillbirth and neonatal death^(13)^. However, some authors also suggest that, given the significant morbidity and mortality associated with placenta accreta spectrum disorders, the use of gadolinium contrast is indicated in some cases, stating that gadolinium adds specificity to the diagnosis, because the interface between the placenta and myometrium is more clearly delineated in contrast-enhanced images^(3)^. Table 1 summarizes the suggested protocol for MRI of the placenta.

**Table 1:** Summary of suggested protocol for MRI of the placenta.

Sequence	Plane	Thickness	Indication
T2-weighted FSE/SSFSE/HASTE	Coronal, sagittal, axial	≤ 4 mm	Placental location, anatomical details, and signs of a placenta accreta spectrum disorder
T2-weighted FIESTA/TrueFISP fat sat	Coronal, sagittal, axial	4 mm	Anatomy, placental margins, and vascularization
T1-weighted 3D fat sat	Axial, sagittal	3 mm	Investigation of bleeding, to identify intraplacental or extraplacental hemorrhage
T2-weighted FSE/SSFSE/ HASTE (focused on the cervix)	Sagittal	4 mm	Study of the cervix, to measure the distance from the placental margin to the internal cervical os
DWI (b = 0/50 and 600/1,000 s/mm^2^)	Axial	≤ 5 mm	Investigation of placental invasion

## MRI FINDINGS IN THE NORMAL PREGNANT UTERUS

**Placenta** - A normal placenta is of uniform thickness, measuring 2-4 cm thick in the middle and gradually decreasing in thickness toward the periphery. At 24-30 weeks of gestation, the placenta exhibits a homogeneous intermediate signal on T2-weighted images and is distinct from the myometrium, which has a hyperintense signal that is more heterogeneous. After week 30, the placenta becomes more heterogeneous, limiting the diagnostic performance of MRI for placenta accreta spectrum disorders^(1)^. A few flow voids (< 5 mm) can be seen in the intraplacental and subplacental regions^(9)^.

**Myometrium** - Up to 30 weeks of gestation, the myometrium usually has a trilaminar appearance, comprising the internal layer, which comprises the decidua basalis and the inner myometrium, forming the uterine-placental interface; the thicker, middle layer; and the external layer, which represents the uterine serosa. The internal and external layers have low signal intensity on T2-weighted images, whereas the thicker middle layer has a high intensity signal in relation to that of the placenta. After week 30, the myometrium begins to thin and the layers become less distinct, being visualized on T2-weighted images as a continuous low-intensity band surrounding the placenta^(1,9)^.

**Figure 1:**
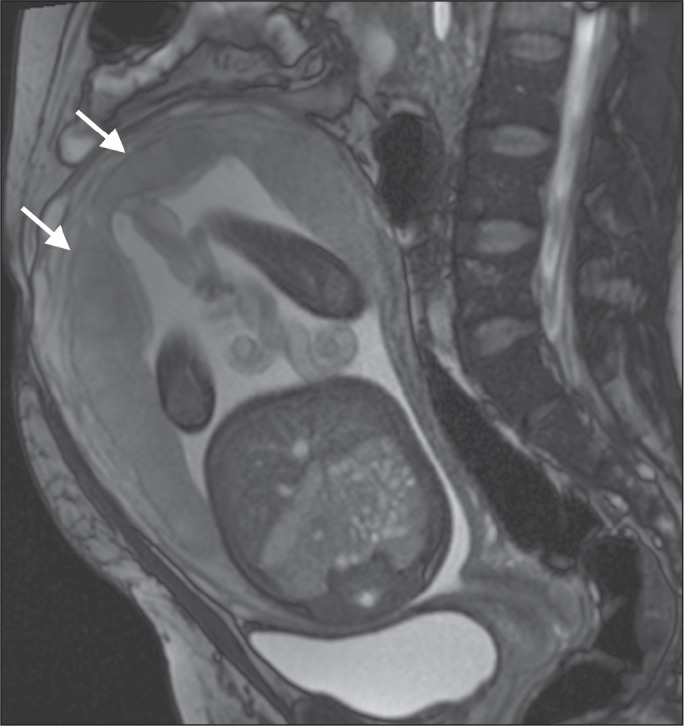
A 30-year-old patient with a normal placenta. Sagittal T2-weighted HASTE sequence showing an inverted pear-shaped uterus and a preserved myometrium-placenta interface (arrows).

**Myometrium-placenta interface** - In T2-weighted MRI sequences, the placenta is usually clearly distinguished from the underlying myometrium by a low-intensity signal (retroplacental line or band) at the myometrium-placenta interface^(9)^, a feature known as the retroplacental T2 dark zone (Figure 1).

**Uterine contour** - As can be seen in Figure 1, a normal uterus is smooth, the fundus and body being wider than the cervix^(9)^.

## MRI FINDINGS IN PLACENTA ACCRETA SPECTRUM DISORDERS

Several MRI features of placenta accreta spectrum disorders have been described in the literature, varying in their sensitivity and specificity. During image interpretation, such findings are not assessed in isolation; using more than one criterion increases diagnostic accuracy^(6,9)^.

**Figure 2:**
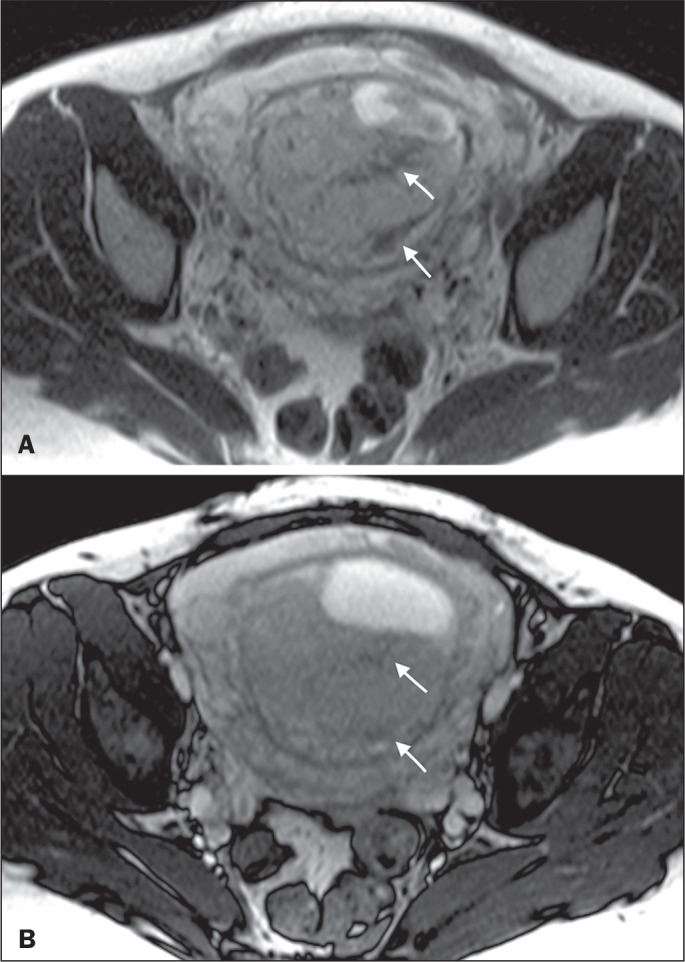
A 30-year-old patient with total placenta previa. Axial T2-weighted HASTE sequence (A) and axial T2-weighted TrueFISP sequence (B), showing dark intraplacental bands (arrows) suggestive of a placenta accreta spectrum disorder.

**Dark intraplacental bands** - On T2-weighted images (Figure 2), areas of low signal intensity that extend across the myometrium-placenta interface are referred to as dark intraplacental bands. These bands are thicker than are the normal placental septa and are distributed randomly^(1,6,9)^.

**Figure 3:**
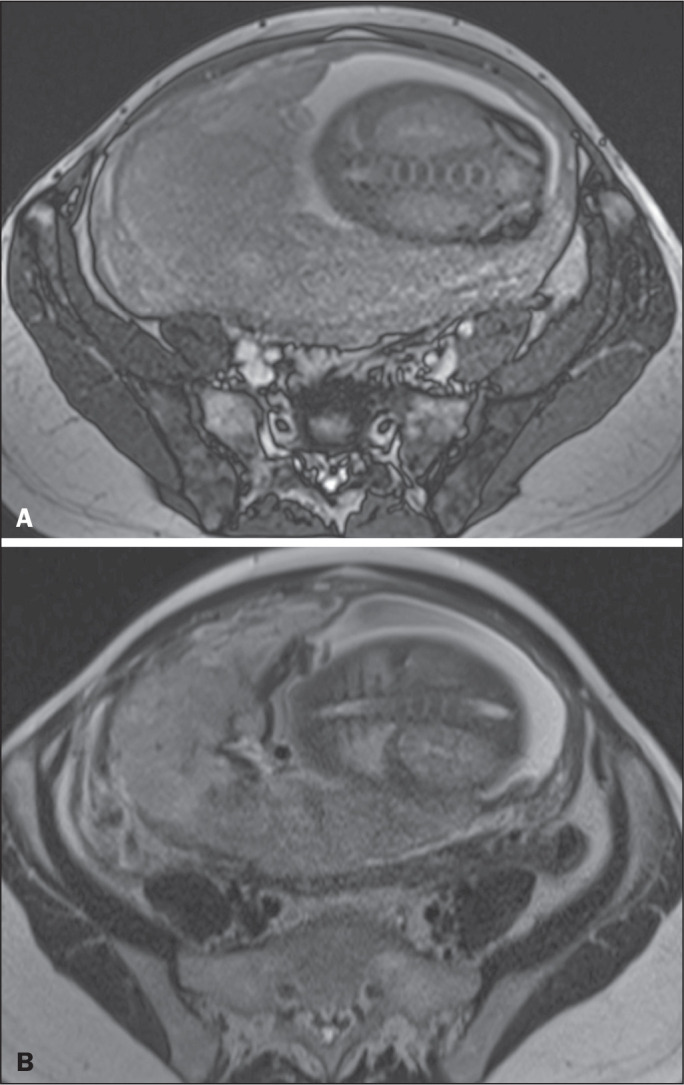
A 36-year-old patient with a placenta accreta spectrum disorder. Axial T2-weighted TrueFISP sequence (A) and axial T2-weighted HASTE sequence (B), showing a diffusely heterogeneous placenta.

**Heterogeneous placenta** - A heterogeneous placenta is caused by the interaction among hemorrhage, dark intraplacental bands, and deep flow voids (Figure 3). A homogeneous placenta can exclude abnormal placentation with high levels of confidence. A mild to moderate degree of heterogeneous signal intensity is considered to be of limited utility as a sign of a placenta accreta spectrum disorder and is typically seen in the third trimester of pregnancy. This sign is relatively nonspecific, because its evaluation is subjective^(1,6,9)^.

**Figure 4:**
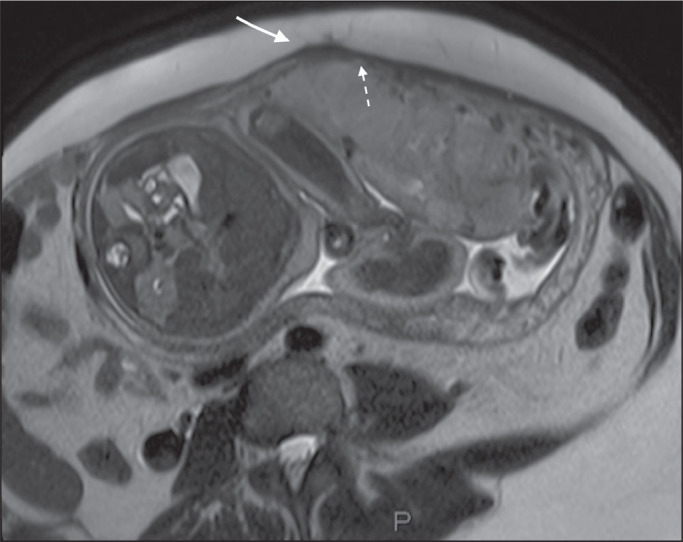
A 36-year-old patient with placenta percreta. Axial T2-weighted HASTE sequence showing abnormal uterine bulging, with a lumpy external uterine contour anteriorly (full arrow), together with myometrial thinning (dashed arrow).

**Abnormal uterine bulging** - Among the MRI findings seen in isolation, some authors consider abnormal uterine bulging to be the most useful sign of a placenta accreta spectrum disorder^(15)^. There are two forms of uterine bulging: diffuse, resulting in a loss of the typical inverted pear shape of the uterus, which takes on an hourglass shape (as can be seen in normal pregnancies); and a focal bulge in the myometrium (Figure 4), which has been reported to be more useful for the diagnosis of a placenta accreta spectrum disorder. The presence of uterine bulging is associated with deeper myometrial invasion^(9,15)^.

**Figure 5:**
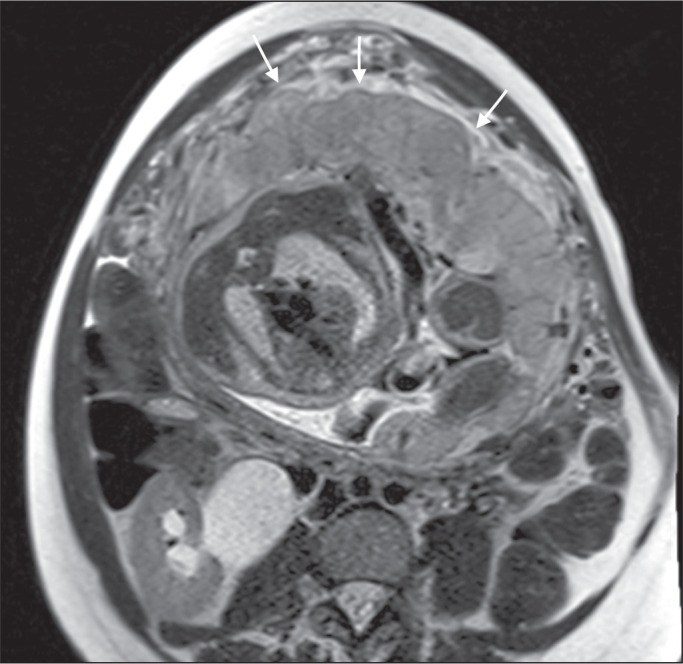
A 35-year-old patient with lobulated placenta (arrows). Coronal T2-weighted HASTE sequence.

**Irregular contour and rounded edge** - On MRI of the placenta, irregular contours and rounded edges are imaging features that are suggestive of placenta accreta spectrum disorders (Figure 5). These findings are frequently observed in conjunction with uterine bulging^(1,6,9)^.

**Figure 6:**
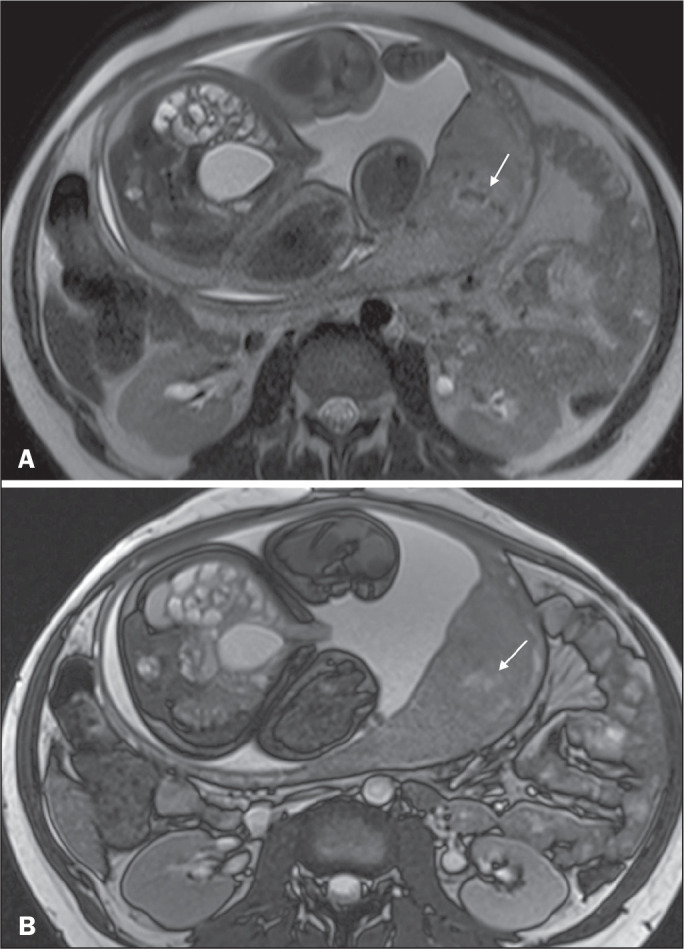
A 33-year-old patient. Axial T2-weighted HASTE and TrueFISP sequences (A and B, respectively), showing prominent intraplacental vessels (arrows), suggestive of a placenta accreta spectrum disorder.

**Figure 7:**
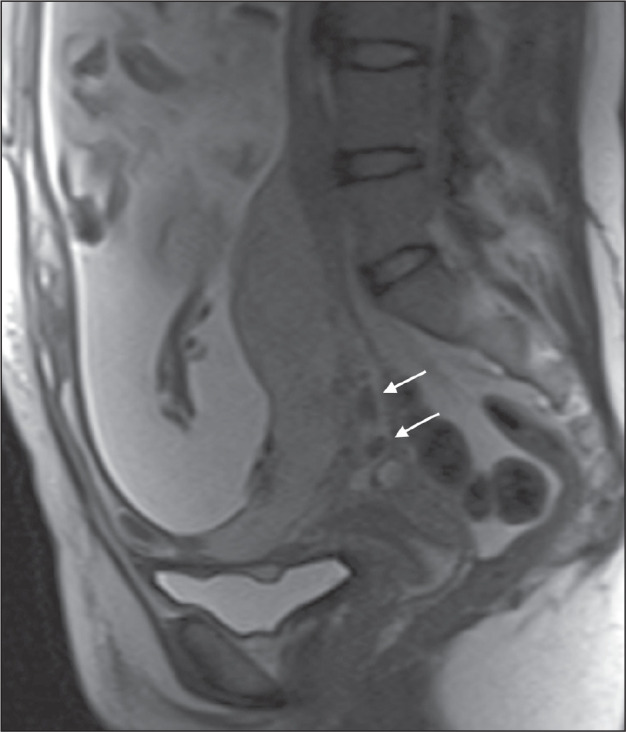
A 30-year-old patient with total placenta previa. Sagittal T2-weighted HASTE sequence showing prominent retroplacental vessels (arrows) at the level of the isthmus and posterior body of the uterus, suggestive of a placenta accreta spectrum disorder.

**Figure 8:**
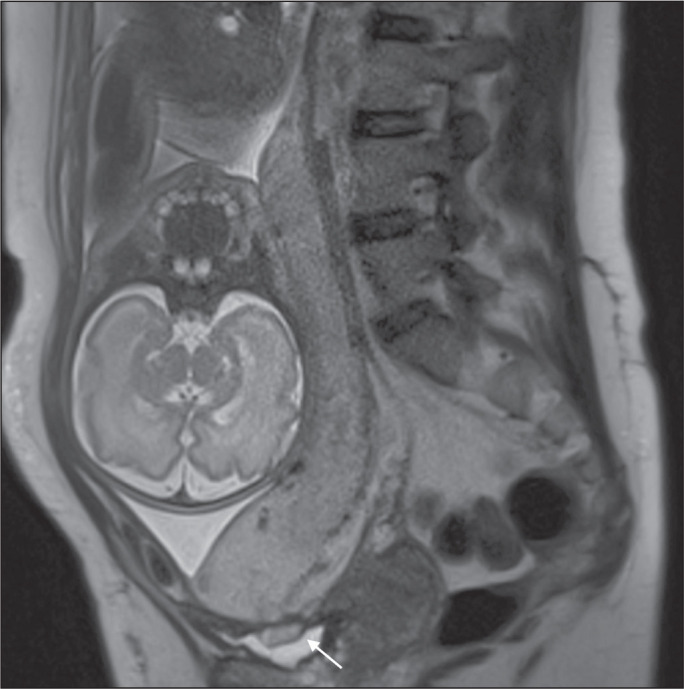
A 30-year-old patient with total placenta previa. Sagittal T2-weighted HASTE sequence showing prominent subplacental vessels, especially at the myometrium-bladder interface (arrow), suggestive of a placenta accreta spectrum disorder.

**Abnormal or disorganized intraplacental and subplacental vascularization** - On MRI, abnormal placental vascularization manifests as dilated tortuous flow voids (> 6 mm) in T2-weighted half-Fourier acquisition single-shot turbo spin-echo (HASTE) sequences and high signal intensity in TrueFISP sequences (Figure 6), often in close proximity to dark intraplacental bands on T2-weighted images and occasionally extending beyond the placenta. Subplacental vascularization may cross the uterine serosa and can be accompanied by extensive neovascularization around the uterus, cervix, vagina, and bladder^(9)^, as depicted in Figures 7 and 8.

**Figure 9:**
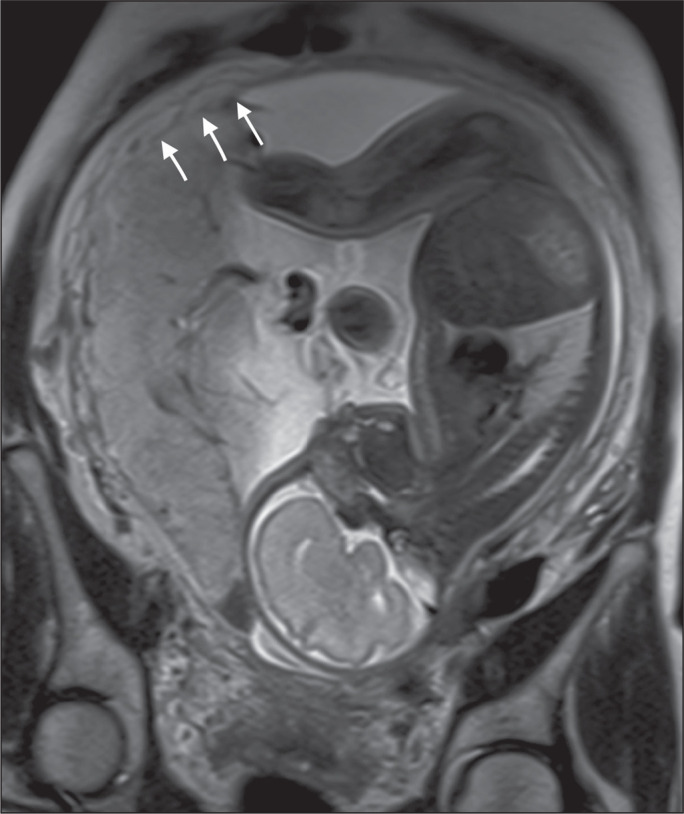
A 39-year-old patient. Coronal T2-weighted HASTE sequence showing retroplacental areas of low-intensity signal halo loss (arrows), together with myometrial thinning.

**Thinning or loss of the retroplacental T2 dark zone** - In cases of placenta accreta spectrum disorders, T2-weighted images show interruption of the myometrium-placenta interface (Figure 9).

**Myometrial thinning** - On MRI, myometrial thinning is the first sign to suggest a placenta accreta spectrum disorder. The myometrium may be as thin as 1 mm in the area of placental insertion and becomes imperceptible in placenta accreta spectrum disorders (Figure 9). This sign has low sensitivity and specificity for a placenta accreta spectrum disorder, because of the physiological thinning of the myometrium that occurs as the pregnancy progresses, especially at the site of a cesarean scar^(9)^.

**Figure 10:**
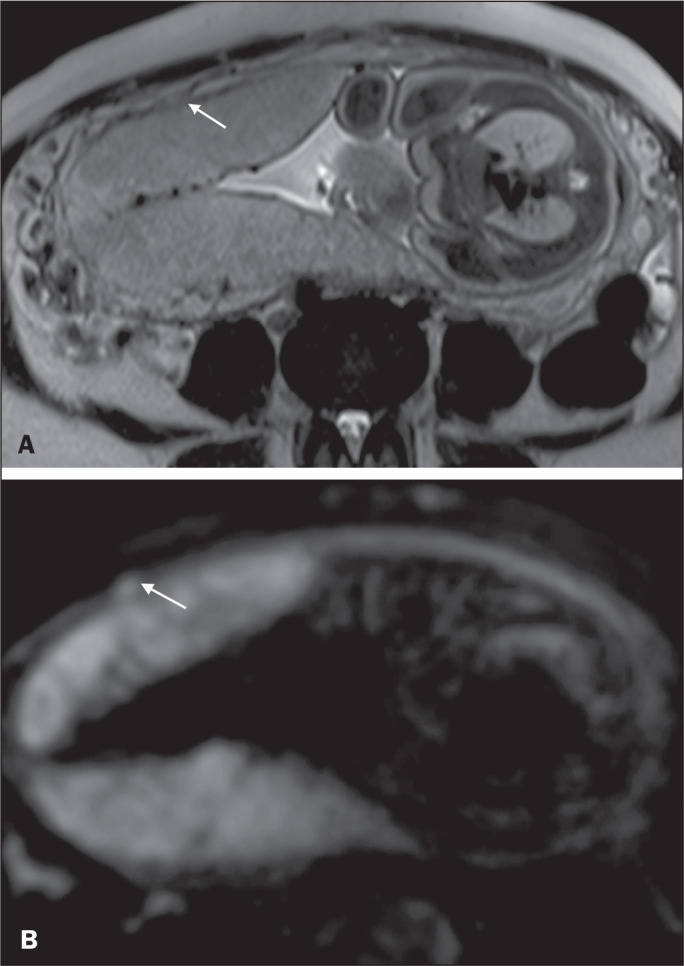
A 39-year-old patient. Axial T2-weighted HASTE and DWI sequences (A and B, respectively), showing a focal rupture of the myometrium (arrow).

**Focal disruption of the myometrium** - Focal disruption of the myometrium at the site of placental invasion (Figure 10) is an MRI feature that can be observed only when the myometrium is well represented^(9)^. Alamo et al.^(16)^ suggested that this sign is the second most common criterion in cases of placental invasion, with a sensitivity of 91%^(16)^.

## MRI FINDINGS IN EXTRAUTERINE TISSUE (PLACENTA PERCRETA)

**Figure 11:**
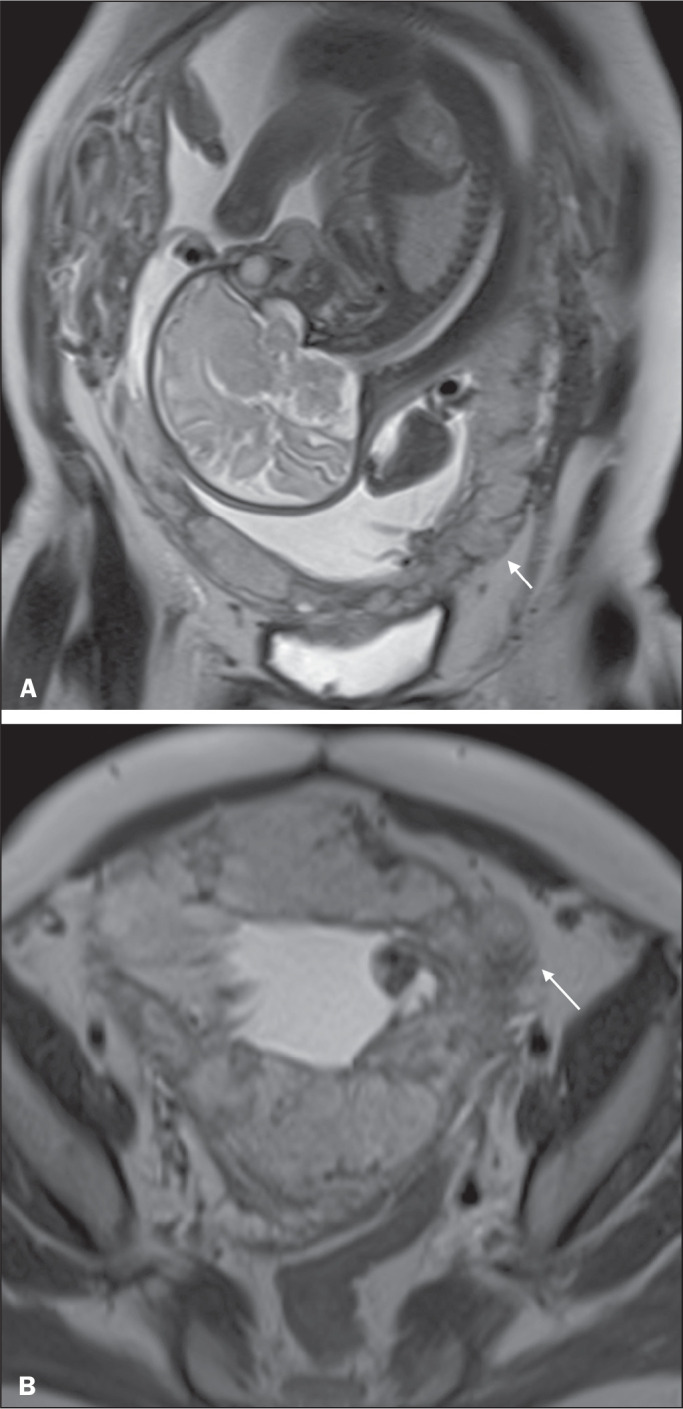
A 35-year-old patient. Coronal T2-weighted HASTE sequence (A) and axial T2-weighted HASTE sequence (B), showing lobulation of the external contour of the uterus in the left anterolateral wall of the inferior portion, with signs of extrauterine extension (arrows), suggestive of placenta percreta.

In general, it is not necessary to distinguish between placenta accreta and placenta increta, because the treatment is the same for both. However, in cases of placenta percreta, the invasion of adjacent organs affects the surgical management, and, on MRI, an attempt should be made to identify the structures involved (Figure 11). Uterine bulging with an irregular placental contour is more evident in placenta percreta than in placenta accreta and placenta increta^(1)^. However, a definitive MRI diagnosis of placenta percreta requires additional findings, such as a total loss of myometrial thickness; obliteration of the adipose plane between the placental tissue and adjacent organs; and interruption of the hypointense line of the bladder, intestinal wall, or the muscles of the abdominal wall/pelvic floor on T2-weighted images^(1)^. Additional criteria for extension to the bladder include inclination of the bladder dome and chaotic vascularization at its interface with the uterus^(9)^.

In the case of placental invasion of adjacent organs, MRI is preferable to ultrasound because it provides a wider field of view, which improves surgical planning^(9)^. Table 2 summarizes the MRI findings for each degree of invasion.

**Table 2:** MRI findings by the degree of invasion: placenta accreta, increta, and percreta.

Extent of placental invasion	MRI findings
Accreta	Loss of the myometrium-placenta interface
	Dark intraplacental bands
	Heterogeneity
	Focal bulging of the uterine contour
Increta	All of the findings listed above Uterine bulging
Percreta	All of the findings listed above
	Abnormal placental vascularization seen as vessels crossing the serosa
	Direct invasion into and beyond the serosa
	Involvement of adjacent structures such as the bladder, rectum, and abdominal wall

## DIAGNOSTIC PITFALLS

**Figure 12:**
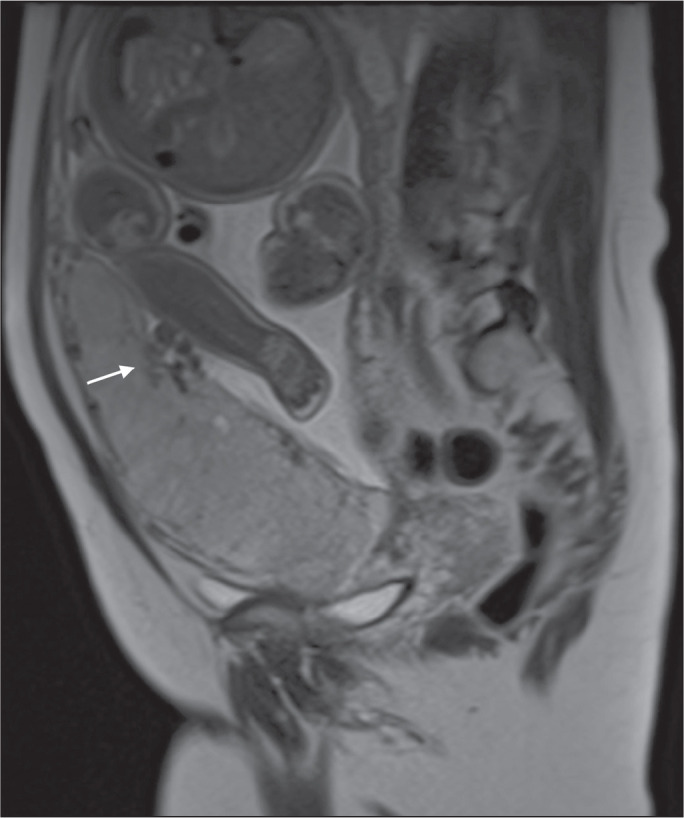
A 39-year-old patient with no signs of placenta accreta. Axial T2-weighted HASTE sequence showing normal intraplacental flow voids near the umbilical cord insertion site (arrow).

**Placental vascularization** - On MRI, some normal flow voids (< 6 mm) can be identified in the subplacental and intraplacental regions, usually at the umbilical cord insertion site^(1,9)^, as depicted in Figure 12.

**Dark intraplacental bands** - After week 30 of gestation, dark intraplacental bands can be observed in the normal placenta, usually on the fetal side of the placenta, although abnormal bands are typically seen on the maternal side^(1,9)^. Such bands can also be seen in pregnant women with placental infarction or an intervillous thrombus^(9)^.

**Bladder varices -** Bladder varices can mimic focal uterine bulging. In such cases, DWI is useful, showing low signal intensity for the bladder varices and high signal intensity for the uterine bulging^(1,9)^.

**Figure 13:**
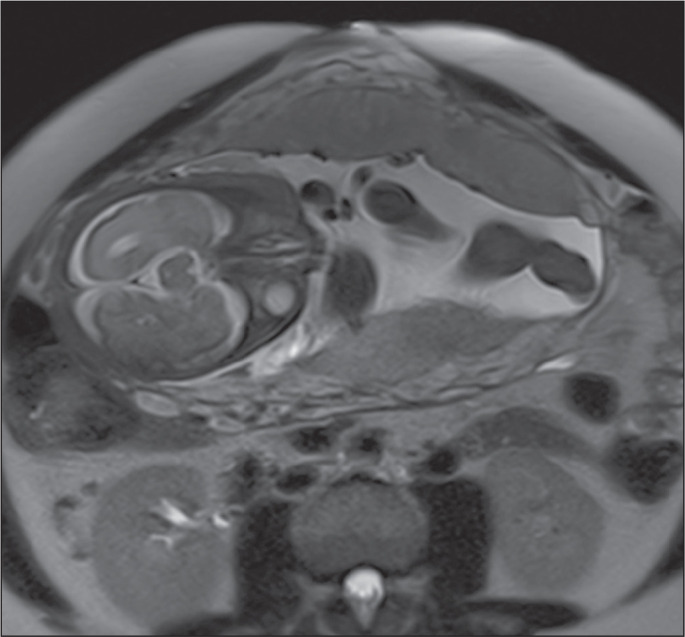
A 38-year-old patient with no signs of placenta accreta. Axial T2-weighted HASTE sequence showing uterine bulging in the umbilicus due to abdominal diastasis.

**Focal bulge in the umbilicus** - At the end of the third trimester of pregnancy, the rectus abdominis sheath may separate and cause a focal bulge of the anterior aspect of the myometrium^(9)^, as shown in Figure 13.

**Loss of the retroplacental T2 dark zone** - On T2-weighted MRI sequences acquired in the early stages of a normal pregnancy, the retroplacental zone of low signal intensity is often absent^(1,9)^.

## CONCLUSION

Placenta accreta spectrum disorders have become more frequent. The use of MRI plays an important role in the prenatal diagnosis of and treatment planning for such disorders. The treatment plan should be carried out by an experienced multidisciplinary team, in order to minimize maternal morbidity and mortality.
